# Testing for HCV Infection: An Update of Guidance for Clinicians and Laboratorians

**Published:** 2013-05-10

**Authors:** Jane P. Getchell, Kelly E. Wroblewski, Alfred DeMaria, Christine L. Bean, Monica M. Parker, Mark Pandori, D. Robert Dufour, Michael P. Busch, Mark E. Brecher, William A. Meyer, Rick L. Pesano, Chong-Gee Teo, Geoffrey A. Beckett, Aufra C. Araujo, Bernard M. Branson, Jan Drobeniuc, Rikita Hatia, Scott D. Holmberg, Saleem Kamili, John W. Ward

**Affiliations:** Assn of Public Health Laboratories; Massachusetts Dept of Public Health; New Hampshire Dept of Health; New York State Dept of Health; San Francisco Dept of Public Health; VA Medical Center, Washington, DC; Blood Systems Inc; LabCorp; Quest Diagnostics; National Center for HIV/AIDS, Viral Hepatitis, STD, and TB Prevention, CDC

In the United States, an estimated 4.1 million persons have been infected with hepatitis C virus (HCV), of whom an estimated 3.2 (95% confidence interval [CI] = 2.7–3.9) million are living with the infection (1). New infections continue to be reported particularly among persons who inject drugs and persons exposed to HCV-contaminated blood in health-care settings with inadequate infection control (2).

Since 1998, CDC has recommended HCV testing for persons with risks for HCV infection (3). In 2003, CDC published guidelines for the laboratory testing and result reporting of antibody to HCV (4). In 2012, CDC amended testing recommendations to include one-time HCV testing for all persons born during 1945–1965 regardless of other risk factors (1).

CDC is issuing this update in guidance because of 1) changes in the availability of certain commercial HCV antibody tests, 2) evidence that many persons who are identified as reactive by an HCV antibody test might not subsequently be evaluated to determine if they have current HCV infection (5), and 3) significant advances in the development of antiviral agents with improved efficacy against HCV (6). Although previous guidance has focused on strategies to detect and confirm HCV antibody (3,4), reactive results from HCV antibody testing cannot distinguish between persons whose past HCV infection has resolved and those who are currently HCV infected. Persons with current infection who are not identified as currently infected will not receive appropriate preventive services, clinical evaluation, and medical treatment. Testing strategies must ensure the identification of those persons with current HCV infection.

This guidance was written by a workgroup convened by CDC and the Association of Public Health Laboratories (APHL), comprising experts from CDC, APHL, state and local public health departments, and academic and independent diagnostic testing laboratories, in consultation with experts from the Veterans Health Administration and the Food and Drug Administration (FDA). The workgroup reviewed laboratory capacities and practices relating to HCV testing, data presented at the CDC 2011 symposium on identification, screening and surveillance of HCV infection (7), and data from published scientific literature on HCV testing. Unpublished data from the American Red Cross on validation of HCV antibody testing also were reviewed.

## Changes in HCV Testing Technologies

Since the 2003 guidance was published (4), there have been two developments with important implications for HCV testing:

1. Availability of a rapid test for HCV antibody. The OraQuick HCV Rapid Antibody Test (OraSure Technologies) is a rapid assay for the presumptive detection of HCV antibody in fingerstick capillary blood and venipuncture whole blood. Its sensitivity and specificity are similar to those of FDA–approved, laboratory-conducted HCV antibody assays (8). In 2011, a Clinical Laboratory Improvements Amendments waiver was granted to the test by FDA. The waiver provides wider testing access to persons at risk for HCV infection, permitting use of the assay in nontraditional settings such as physician offices, hospital emergency departments, health department clinics, and other freestanding counseling and testing sites.
2. Discontinuation of RIBA HCV. The Chiron RIBA HCV 3.0 Strip Immunoblot Assay (Novartis Vaccines and Diagnostics) that was recommended (4) for supplemental testing of blood samples after initial HCV antibody testing is no longer available. As a result, the only other FDA-approved supplemental tests for HCV infection are those that detect HCV viremia.

## Identifying Current HCV Infections

In 2011, FDA approved boceprevir (Victrelis, Merck & Co.) and telaprevir (Incivek, Vertex Pharmaceuticals) for treatment of chronic hepatitis C genotype 1 infection, in combination with pegylated interferon and ribavirin, in adult patients with compensated liver disease. Boceprevir and telaprevir interfere directly with HCV replication. Persons who complete treatment using either of these drugs combined with pegylated interferon and ribavirin are more likely to clear virus (i.e., have virologic cure), compared to those given standard therapy based on pegylated interferon and ribavirin (9). Viral clearance, when sustained, stops further spread of HCV and is associated with reduced risk for hepatocellular carcinoma (10) and all-cause mortality (11). Other compounds under study in clinical trials hold promise for even more effective therapies (6).

Because antiviral treatment is intended for persons with current HCV infection, these persons need to be distinguished from persons whose infection has resolved. HCV RNA in blood, by nucleic acid testing (NAT), is a marker for HCV viremia and is detected only in persons who are currently infected. Persons with reactive results after HCV antibody testing should be evaluated for the presence of HCV RNA in their blood.

## Benefits of Testing for Current HCV Infection

Accurate testing to identify current infection is important to 1) help clinicians and other providers correctly identify persons infected with HCV, so that preventive services, care and treatment can be offered; 2) notify tested persons of their infection status, enabling them to make informed decisions about medical care and options for HCV treatment, take measures to limit HCV-associated disease progression (e.g., avoidance or reduction of alcohol intake, and vaccination against hepatitis A and B), and minimize risk for transmitting HCV to others; and 3) inform persons who are not currently infected of their status and the fact that they are not infectious.

## Recommended Testing Sequence

The testing sequence in this guidance is intended for use by primary care and public health providers seeking to implement CDC recommendations for HCV testing (1,3,4). In most cases, persons identified with HCV viremia have chronic HCV infection. This testing sequence is not intended for diagnosis of acute hepatitis C or clinical evaluation of persons receiving specialist medical care, for which specific guidance is available (12).

Testing for HCV infection begins with either a rapid or a laboratory-conducted assay for HCV antibody in blood (Figure). A nonreactive HCV antibody result indicates no HCV antibody detected. A reactive result indicates one of the following: 1) current HCV infection, 2) past HCV infection that has resolved, or 3) false positivity. A reactive result should be followed by NAT for HCV RNA. If HCV RNA is detected, that indicates current HCV infection. If HCV RNA is not detected, that indicates either past, resolved HCV infection, or false HCV antibody positivity.

### Initial Testing for HCV Antibody

An FDA-approved test for HCV antibody should be used. If the OraQuick HCV Rapid Antibody Test is used, the outcome is reported as reactive or nonreactive. If a laboratory-based assay is used, the outcome is reported as reactive or nonreactive without necessarily specifying signal-to-cutoff ratios.

### Testing for HCV RNA

An FDA-approved NAT assay intended for detection of HCV RNA in serum or plasma from blood of at-risk patients who test reactive for HCV antibody should be used. There are several possible operational steps toward NAT after initial testing for HCV antibody:

1. Blood from a subsequent venipuncture is submitted for HCV NAT if the blood sample collected is reactive for HCV antibody during initial testing.
2. From a single venipuncture, two specimens are collected in separate tubes: one tube for initial HCV antibody testing; and a second tube for HCV NAT if the HCV antibody test is reactive.
3. The same sample of venipuncture blood used for initial HCV antibody testing, if reactive, is reflexed to HCV NAT without another blood draw for NAT (13).
4. A separate venipuncture blood sample is submitted for HCV NAT if the OraQuick HCV Rapid Antibody Test for initial testing of HCV antibody has used fingerstick blood.

## Supplemental Testing for HCV Antibody

If testing is desired to distinguish between true positivity and biologic false positivity for HCV antibody, then, testing may be done with a second HCV antibody assay approved by FDA for diagnosis of HCV infection that is different from the assay used for initial antibody testing. HCV antibody assays vary according to their antigens, test platforms, and performance characteristics, so biologic false positivity is unlikely to be exhibited by more than one test when multiple tests are used on a single specimen (14).

## Test Interpretation and Further Action

See Table.

## Laboratory Reporting

“Acute hepatitis C” and “hepatitis C (past or present)” are nationally notifiable conditions, and are subject to mandated reporting to health departments by clinicians and laboratorians, as determined by local, state or territorial law and regulation. Surveillance case definitions are developed by the Council of State and Territorial Epidemiologists in collaboration with CDC (15). In all but a few jurisdictions, positive results from HCV antibody and HCV RNA testing that are indicative of acute, or past or present HCV infection, are reportable. Specific policies for laboratory reporting are found at health department websites (16).

## Future Studies

Research, development, validation, and cost-effectiveness studies are ongoing to inform the best practices for detecting HCV viremia and for distinguishing between resolved HCV infection and biologic false positivity for HCV antibody in persons in whom HCV RNA is not detected. Outcomes of these studies will provide comprehensive guidance on testing, reporting, and clinical management, and will improve case definitions for disease notification and surveillance.

## Figures and Tables

**FIGURE:**
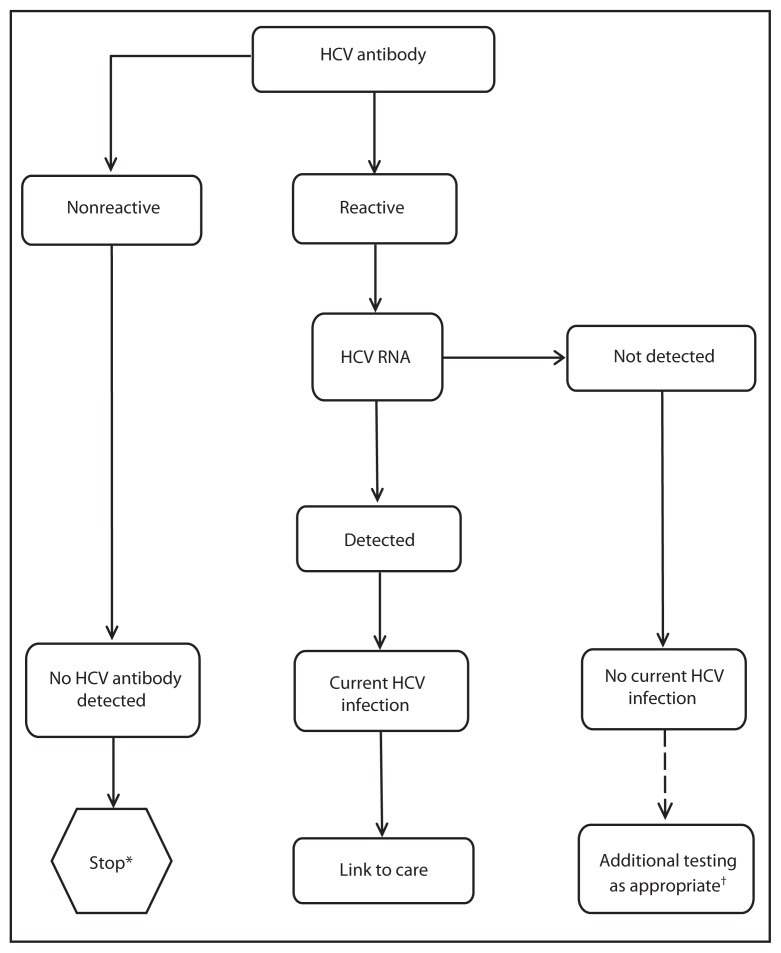
Recommended testing sequence for identifying current hepatitis C virus (HCV) infection ^*^ For persons who might have been exposed to HCV within the past 6 months, testing for HCV RNA or follow-up testing for HCV antibody is recommended. For persons who are immunocompromised, testing for HCV RNA can be considered. ^†^ To differentiate past, resolved HCV infection from biologic false positivity for HCV antibody, testing with another HCV antibody assay can be considered. Repeat HCV RNA testing if the person tested is suspected to have had HCV exposure within the past 6 months or has clinical evidence of HCV disease, or if there is concern regarding the handling or storage of the test specimen.

**TABLE:** Interpretation of results of tests for hepatitis C virus (HCV) infection and further actions

Test outcome	Interpretation	Further action
HCV antibody nonreactive	No HCV antibody detected	Sample can be reported as nonreactive for HCV antibody. No further action required. If recent HCV exposure in person tested is suspected, test for HCV RNA.*
HCV antibody reactive	Presumptive HCV infection	A repeatedly reactive result is consistent with current HCV infection, or past HCV infection that has resolved, or biologic false positivity for HCV antibody. Test for HCV RNA to identify current infection.
HCV antibody reactive, HCV RNA detected	Current HCV infection	Provide person tested with appropriate counseling and link person tested to medical care and treatment.†
HCV antibody reactive, HCV RNA not detected	No current HCV infection	No further action required in most cases. If distinction between true positivity and biologic false positivity for HCV antibody is desired, and if sample is repeatedly reactive in the initial test, test with another HCV antibody assay. In certain situations§ follow up with HCV RNA testing and appropriate counseling.

* If HCV RNA testing is not feasible and person tested is not immunocompromised, do follow-up testing for HCV antibody to demonstrate seroconversion. If the person tested is immunocompromised, consider testing for HCV RNA.

† It is recommended before initiating antiviral therapy to retest for HCV RNA in a subsequent blood sample to confirm HCV RNA positivity.

§ If the person tested is suspected of having HCV exposure within the past 6 months, or has clinical evidence of HCV disease, or if there is concern regarding the handling or storage of the test specimen.
